# Ras signalling in pathogenic yeasts

**DOI:** 10.15698/mic2018.02.612

**Published:** 2017-12-18

**Authors:** Daniel R. Pentland, Elliot Piper-Brown, Fritz A. Mühlschlegel, Campbell W. Gourlay

**Affiliations:** 1 Kent Fungal Group, School of Biosciences, University of Kent, Canterbury, Kent, United Kingdom, CT2 7NJ.; 2 Laboratoire national de santé, 1, Rue Louis Rech, L-3555 Dudelange, Luxembourg.

**Keywords:** Ras signalling, C. albicans, C. neoformans, pathogenicity, morphogenesis, biofilm

## Abstract

The small GTPase Ras acts as a master regulator of growth, stress response and cell death in eukaryotic cells. The control of Ras activity is fundamental, as highlighted by the oncogenic properties of constitutive forms of Ras proteins. Ras also plays a crucial role in the pathogenicity of fungal pathogens where it has been found to regulate a number of adaptions required for virulence. The importance of Ras in fungal disease raises the possibility that it may provide a useful target for the development of new treatments at a time when resistance to available antifungals is increasing. New findings suggest that important regulatory sequences found within fungal Ras proteins that are not conserved may prove useful in the development of new antifungals. Here we review the roles of Ras protein function and signalling in the major human yeast pathogens *Candida albicans* and *Cryptococcus neoformans *and discuss the potential for targeting Ras as a novel approach to anti-fungal therapy.

## RAS PROTEINS - FORM AND FUNCTION

The Ras superfamily consists of small G-binding proteins that have been divided into five main groups on the basis of sequence and functional similarity: Ras, Rho, Arf/Sar, Rab and Ran 1. As with all G proteins, Ras activation is dependent on the exchange of bound GDP for GTP and deactivation via hydrolysis of GTP to GDP. Cycling between active and non-active states is regulated by Guanine nucleotide exchange factors (GEF), dissociation inhibitor’s (GDI) and GTPase Activating Proteins (GAP) accessory proteins 2. The activation and deactivation cycle of Ras proteins couple a range of stimuli to effector proteins and as such these G-proteins serve as regulatory "switches" within a variety of cellular processes. The importance of Ras signalling is highlighted by the dramatic effects that can be observed upon inappropriate activation. For example mutations that lead to the constitutive activation of Ras signalling have been estimated to occur in ~50% of all tumours 3. In addition, Ras proteins play important roles in the regulation of growth and adaption in fungal cells. In this review we will focus on Ras protein function in the major human yeast pathogens *Candida albicans* (*C. albicans*) and *Cryptococcus neoformans *(*C. neoformans*).

## RAS SIGNALLING IN THE FUNGAL PATHOGEN OF HUMANS *C. ALBICANS*

### *C. albicans* virulence and pathogenicity

*C. albicans *is a commensal organism that is commonly found on the mucosal surfaces of the oral cavity, gastrointestinal tract and genitourinary tract of healthy individuals 4,5. However *C. albicans* is also a well characterised opportunistic pathogen 4,6 and a serious health-risk amongst immunocompromised individuals, such as those suffering from HIV infection, or persons living with indwelling medical devices such as catheters or voice prostheses 7,8. The infections caused by *C. albicans* range from superficial infection of mucosal and non-mucosal surfaces (candidiasis) to a full systemic infection (candidaemia) also affecting internal organs 4. Superficial mucosal surface infections can be readily treated with a range of antifungals. For example, vulvovaginal candidiasis (VVC) is usually successfully treated with azole antifungals like fluconazole 9_._ However, candidaemia is associated with a high mortality even when treated with a variety of classes of antifungal agents 10. As with many *C. albicans *infections, weakened immune defences are a significant risk factor for developing candidaemia. In healthy individuals, neutrophils provide suitable defence against *C. albicans*. As such, neutropenia, either as a result of particular blood cancers or treatment with immunosuppressants, significantly increases the risk of developing candidaemia. Furthermore, damage to the mucosa of the gastrointestinal tract, for example due to surgery, is also a risk factor as it enables the spread of *C. albicans* 11. The symptoms of candidaemia range from fever and chills which do not abate following antibiotic treatment to severe sepsis or septic shock similar to that of bacterial septicaemia 9_._ However, a lack of precise symptoms can lead to delayed diagnosis and required antifungal treatment leading to increased mortality 12. It has been reported that even a delay of as little as 12-24h can double mortality rate 13. Due to this, it has been suggested to prophylactically administer antifungals after any event which is likely to increase the risk of candidaemia, such as after abdominal surgery or bone marrow transplant 9. Although the majority of cases of candidiasis and candidaemia are caused by *C. albicans*, there are other species within the *Candida *genus which are also pathogenic in humans. These include *Candida glabrata, Candida tropicalis, Candida dubliniensis and Candida parapsilosis*. *Candida *species have increasingly become associated with nosocomial infections 6; in fact, *C. albicans *is recognised as the fourth most common cause of all hospital-acquired infections in the USA 4.

### Morphogenesis, pathogenicity and Ras signalling in *C. albicans*

**Figure 1:**
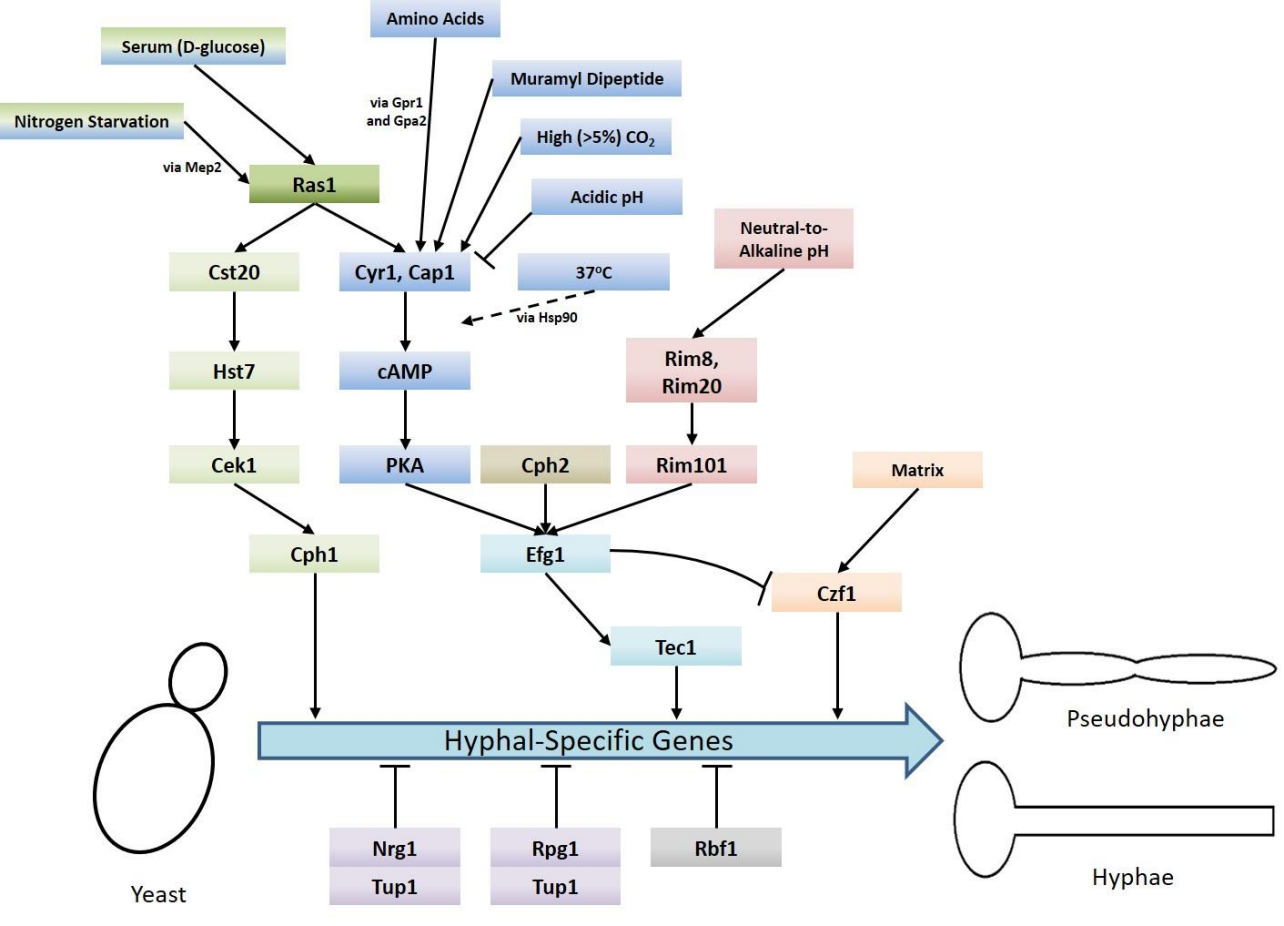
FIGURE 1: Summary of the signalling pathways and stimuli which regulate the yeast-to-hyphae morphogenic switch in *Candida albicans*. Several different pathways are responsible for inducing the yeast-to-hyphae switch: MAP kinase pathway (green), Cyr1-PKA pathway (blue), pH response pathway (red), and matrix response pathway (orange). There are also two pathways which negatively regulate the switch: Tup1-Nrg1-Rpg1 pathway (purple) and Rbf1 pathway (grey). The signalling pathways that engaged in response to different conditions to regulate the yeast-to-hyphae morphogenic switch in *C. albicans* are also indicated.

An important aspect of *C. albicans *biology, in terms of pathogenesis, is its ability to undergo morphogenesis from a yeast, to pseudohyphal or hyphal forms in response to environmental cues. The virulence of *C. albicans* is closely linked with the capacity to switch between these forms. Hyphal *C. albicans *cells are frequently located at sites of tissue invasion, moreover, cells which are unable to readily form hyphae exhibit reduced virulence 4. However as strains that are incapable of growing in the yeast form also have less virulence it has been proposed that both the yeast and hyphal forms play important roles during infection 14,15. Ras signalling is crucial to the integration of environmental cues with morphogenesis and *C. albicans *possesses two Ras genes - *RAS1* and *RAS2*, which encode the Ras1 protein and a highly divergent Ras-like protein termed Ras2 16. The importance of Ras1 signalling to the virulence of *C. albicans* is demonstrated by the fact that mutants which lack Ras1 are defective in their ability to undergo hyphal transition and exhibit reduced virulence in mouse infection models 17. Ras signalling is now known to mediate the induction of hyphal growth in response to a variety of environmental cues including growth at 37^°^C (via alleviation of Hsp90-mediated repression of the Ras1-cAMP-PKA pathway) 18,19, exposure to high levels of CO_2_ 20, N-acetylglucosamine 21 and serum exposure 22. These environmental signals are transduced through the cyclic AMP-protein kinase A and a mitogen-activated protein (MAP) kinase pathways (Figure 1) 4. These pathways culminate in the regulation of transcription factors which control the expression of hyphal-specific genes (HSGs) such as Als3 (adhesin) 23, Hwp1 (invasin) 24, Hyr1 (host immune response modulator) 25, and Hgc1 (hyphal-specific cyclin) 26.

Upon activation, Ras1 directly interacts with and activates Cyr1 (the *C. albicans* adenylate cyclase), causing an increase in the production of the second messenger cAMP 17. cAMP causes the derepression of two isoforms of protein kinase A (PKA) by triggering the dissociation of the PKA regulatory subunit (Bcy1) from the catalytic subunits (Tpk1 or Tpk2). The activation of PKA stimulates several processes within the cell including the yeast-to-hyphae switch 27. PKA is believed to phosphorylate the transcription factor Efg1 on threonine-206, thereby activating it and resulting in the expression of HSGs 28. The Tpk1 and Tpk2 isoforms have some redundant functions in *C. albicans*, however, they also have specific roles in filamentation. For example, Tpk1 is necessary for the expression of genes encoding proteins involved in branched chain amino acid biosynthesis, and Tpk2 negatively regulates iron uptake genes and positively regulates those associated with trehalose degradation and water homeostasis 29.

A number of environmental signals promote the yeast-to-hyphae switch via the Ras1-Cyr1-PKA pathway. Some of these signals, including CO_2_ interface directly with the Cyr1 adenylate cyclase to activate it 20. CO_2_ is able to do this because, unlike most signalling molecules, it is able to enter the cell by simple diffusion and is maintained in the cell as HCO_3_^-^ via conversion by a carbonic anhydrase encoded by *NCE103* 20. It has recently been discovered that the expression of *NCE103* is controlled in response to CO_2_ availability by the bZIP transcription factor Rca1; Rca1 is regulated in a CO_2_-dependent manner by the Sch9 kinase via a cascade mediated by lipid/Pkh1/2 signalling 30. A lysine residue at position 1373 is critical for CO_2_ activation of Cyr1. This lysine residue is located in the C-terminal catalytic domain and makes up a receptor site which detects increased HCO_3_^-^ levels 31, leading to increased cAMP production and activation of PKA filamentation 20. The response of *C. albicans *to CO_2_ is of interest because within a mammalian host, the levels of CO_2_ are approximately 150x that of normal air (~5% compared to 0.03%). It may be the case that high levels of CO_2_, such as are found within the upper respiratory tract, may promote *C. albicans *colonisation. However, it is interesting to note that other pathogenic *Candida* species, including *C. dubliniensis*, *C. glabrata*, *C. paropsilosis *and *C. krusei*, do not undergo the yeast-to-hyphae transition in response to elevated CO_2_ 20. Although this does not rule out the fact that the adenylyl cyclase of the latter species is activated by carbon dioxide/bicarbonate; the physiological significance of CO_2_ sensing with regards to *Candida* infection remains to be determined.

Muramyl dipeptide (MDP), the minimal biologically active subunit of bacterial peptidoglycan, also induces *C. albicans* filamentation by acting directly upon Cyr1 32. A further signal which can cause morphogenesis through direct interaction with Cyr1 are amino acids. Amino acids, when in the presence of glucose, activate Cyr1 via upstream signalling through the G-protein coupled receptor Gpr1 and its Gα protein Gpa2 33. Upon its activation by Gpr1, Gpa2 is believed to bind to a Gα domain on the Cyr1 adenylate cyclase thereby activating it 33. This binding of Gpa2 to a fungal adenylate cyclase Gα domain has been demonstrated in fission yeast 34 but it is yet to be proved experimentally in *C. albicans*.

In contrast, acidic pH causes a reduction in signalling through the Ras1-Cyr1-PKA pathway via a Ras1-independent downregulation of Cyr1 activity 35. *C. albicans *cells grown at pH 4 in hyphae-inducing conditions do not form hyphae, instead remaining as yeast or pseudohyphal cells and this is not reliant upon Ras1. It has also been observed that low extracellular pH results in fast and sustained decreases in intracellular pH which potentially contributes to reduced cAMP signalling through the reduction of intracellular bicarbonate levels 35.

The yeast-to-hyphae switch in response to exposure to serum relies on Ras1 signalling upstream of Cyr1 22. The component of serum principally responsible for the induction of hyphal growth is D-glucose which is able to activate both the Ras1-Cyr1-PKA pathway and the MAP-kinase pathway 22,36. The precise mechanism of Ras1 activation by D-glucose in *C. albicans* remains to be elucidated but in *Saccharomyces cerevisiae *(*S. cerevisiae*) it depends on both an intracellular phosphorylated form of D-glucose and a G-protein coupled receptor Gpr1 with its Gα protein Gpa2 37. Gpr1-type receptors have been characterised in *C. albicans* 33 and so a similar mechanism for Ras1 activation may exist in this pathogen. However, deletion of either CaGpr1 or CaGpa2 had no effect on D-glucose-mediated cAMP signalling, but deletion of CaCdc25 (the *C. albicans *Ras1 GEF) or CaRas1 eliminated this signalling 33. These findings indicate Ras1 activation via Cdc25 is the primary mechanism by which D-glucose induces morphogenesis. The response of *C. albicans* to D-glucose is of physiological relevance because links between candida infection and hyperglycaemia 38 as well as insulin-dependent diabetes mellitus 39 have been reported. Moreover, *C. albicans* cells have increased resistance to oxidative and cationic stresses upon exposure to levels of glucose that may be found in the bloodstream 40.

Ras1 has also been shown to regulate hyphal transition in response to other environmental cues, such as nitrogen starvation 41, via MAP kinase signalling. As with the Cyr1/PKA pathway, the regulatory MAP kinase cascade is also activated by Ras1 17 and consist of the kinases Cst20, Hst7 and Cek1 42,43,44,45,46. Ras1/MAPK signalling culminates in the phosphorylation and activation of the transcription factor Cph1, which in turn promotes the expression of HSGs (Figure 1) 33. It has been shown that inactivation of the Ras1-Cyr1-PKA pathway inhibits filamentous growth in the majority of usual hyphae-inducing conditions, however, inactivation of the MAP-kinase pathway only prevents filamentous growth in only a specific subset of conditions 43.

The CaRas2 protein contains several variations in conserved motifs typically thought to be critical for Ras-related activities and is thus considered an unusual Ras protein. Sequence alignment using BLAST has shown that the *C. albicans *Ras2 protein only has 25-30% identity with all other fungal Ras proteins in the database except a Ras-like protein only found in *Candida dubliniensis* (80% identity) 47. When *RAS2* is deleted in a *ras1*∆/∆ background, intracellular cAMP levels are restored to approximately 30% of wild type levels (*ras1*∆/∆ mutant has a 20x reduction in cAMP). Ras1 and Ras2 may therefore exhibit antagonistic roles in *C. albicans* 47. This is intriguing since the deletion of *RAS2* in a *ras1*∆/∆ background results in a significantly increased defect in hyphal morphogenesis. Nevertheless as *ras2∆*/*∆ *mutants themselves exhibit normal hyphal development 47 the role of Ras2 in morphogenesis and pathogenesis in *C. albicans* has yet to be elucidated.

In addition to the signalling pathways which drive the yeast-to-hyphae switch, there are also negative regulators that are controlled by Ras signalling (Figure 1). Hyphal-specific genes are repressed by the global-repressor Tup1 48 via the specific DNA-binding proteins Nrg1 49,50 and Rfg1 51; the deletions of each of these three proteins results in *C. albicans *cells which are constitutively hyphal even under non-hyphal inducing conditions 48,49,50,51. Approximately half of the genes found to be upregulated during hyphal development in response to 37^°^C and serum are repressed by Tup1 and Nrg1 or Rfg1, suggesting that repression removal is a crucial step in the yeast-to-hyphae switch 52. Consistently, it has been found that hyphal-inducing conditions such as serum exposure and growth at 37^°^C cause a reduction in the expression levels of *NRG1*, leading to the conclusion that one way in which repression of hyphal-specific genes is overcome during the yeast-to-hyphae switch is via down-regulation of the repressors 50. Ras1-Cyr1-PKA pathway activation results in the prompt but short-term removal of Nrg1 from the promoters of hyphal-specific genes. The maintenance of this repression elimination, and hence hyphal development, is achieved through the subsequent recruitment of the Hda1 histone deacetylase which deacetylates a subunit of NuA4 histone acetyltransferase, causing it to also be removed from the promoter. This results in the coiling of the portion of the promoter containing the Nrg1 binding site, preventing the re-binding of Nrg1. It is important to note the removal of Nrg1 is an absolute prerequisite for the Hda1 recruitment 53.

### Ras signalling and white-opaque switching in *C. albicans*

*C. albicans* was traditionally considered to be asexual, only existing as an obligate diploid 54. However, it has now been discovered that mating occurs between homozygous diploid mating type-like (*MTL*) **a **and α strains in this organism, producing an **a**/α tetraploid product 54,55 which then undergoes ‘concerted chromosome loss’ to form diploid progeny 56. *C. albicans* has also been reported to have a viable haploid state which can mate to restore the diploid form 57. While this is similar to the mating program in *S. cerevisiae* (two haploid mating types; **a **and α which combine to generate an **a**/α diploid product) 58 it differs in several key respects.

Mating in *C. albicans* is reliant on a reversible phenotypic switch between two states termed ‘white’ and ‘opaque’. Only the ‘opaque’ state is capable of mating efficiently; ‘opaque’ cells have been demonstrated to mate approximately 10^6^ fold more readily than ‘white’ cells 59. ‘White’ cells are fairly round and form white, dome-shaped colonies on solid agar, they also express a specific set of genes. In contrast, ‘opaque’ cells tend to be larger and more oblong, forming darker colonies which grow flatter against solid agar. ‘Opaque’ cells also express a specific set of genes which differ from those expressed in ‘white’ cells 60.

This unusual mating program involving a reversible phenotypic switch hitherto seems to be unique to *C. albicans* as well as the very closely related fungal species *C. dubliniensis* 61. It appears that ‘white’ cells are better suited for growth and survival within a mammalian host. Therefore, it is likely this unusual mating program has evolved to allow *C. albicans *to survive the variety of environments within a mammalian host while still being able to produce mating-competent cells 59.

Only *C. albicans *cells which are homozygous at the *MTL* locus (**a**/**a **or α/α) are capable of reversibly switching between ‘white’ and ‘opaque’ states, and are thus capable of efficient mating 55,62. This is because two homeodomain proteins called Mtl**a**1 and Mtlα2, encoded by the *MTL****a*** and *MTLα* alleles respectively, work together to inhibit white-opaque switching 59. These proteins are both present in *MTL* heterozygous cells (**a**/α) and thus white-opaque switching cannot occur, only one of these two proteins is present in *MTL* homozygous cells, meaning white-opaque switching is not suppressed and mating (between **a**/**a **cells and α/α cells) can take place.

The transcription factor Wor1 is the master regulator of the white-opaque switch and acts in an all-or-nothing manner; it is virtually undetectable in white cells but highly expressed in opaque cells (expression is approximately 47-fold higher in opaque cells) 63. Wor1 has been shown to control the expression of its own gene *WOR1 *in either a positive feedback or double-negative feedback loop and drives the *C. albicans* cell into the opaque state 64. Due to *WOR1* being repressed by the Mtl**a**1 and Mtlα2 proteins 63, Wor1 is not present in *MTL* heterozygous cells (**a**/α) cells and ectopic *WOR1* expression in these cells causes them to undergo the white-opaque switch 64.

The Ras1-Cyr1-PKA pathway is known to have a role in the white-opaque switch. High levels of CO_2 _can induce this switch; in 20% CO_2 _the switch has been reported to occur with up to 105x more frequency compared to normal air 65. In a *ras1*Δ/Δ mutant and a *cdc35*Δ/Δ mutant (which lacks the CO_2_-responsive Cyr1 adenylate cyclase) white-opaque switching is reduced in both normal air and 1% CO_2_ compared to wild-type but normal in 20% CO_2_ 65. This suggests signalling through the Ras1-Cyr1-PKA pathway is important for the switch in normal air and moderate CO_2_ but not in very high levels of CO_2_.

High N-acetylglucosamine levels also induce the white-opaque switch, and this switch in response to N-acetylglucosamine is significantly diminished from 90.5±3.8% cells in the wild-type to 11.2±1.5% in a *ras1*Δ/Δ mutant 66. Likewise, in a *cdc35*Δ/Δ mutant, 8.0±3.5% of cells undergo the white-opaque response in the presence of N-acetylglucosamine compared to 86.9±4.3% in the wild-type 66. These results suggest the N-acetylglucosamine switch occurs via signalling through the Ras1-Cyr1-PKA pathway. Furthermore, when the master switch regulator Wor1 is overexpressed in a *ras1*Δ/Δ, *cdc35*Δ/Δ, *tpk1*Δ/Δ or *tpk2*Δ/Δ background, the cells are driven into the opaque state. Conversely, a *wor1*Δ/Δ mutant does not switch in the presence of N-acetylglucosamine 66. Wor1 contains a consensus PKA phosphorylation motif with a phosphorylatable threonine at residue 67 64, and it has been demonstrated that this threonine is absolutely required for white-opaque switching in response to N-acetylglucosamine 66. These results imply that the transcription factor Wor1 functions downstream of the Ras1-Cyr1-PKA pathway to induce white-opaque switching in the presence of N-acetylglucosamine.

In addition to Wor1 there are also other transcription factors which act as switch regulators, specifically; Efg1 (itself Ras1-regulated), Czf1, Wor2 67 and Wor3 68. Binding sites for Wor1 have been identified upstream of *EFG1, CZF1 *and *WOR2*, indicating that these transcription factors function in a regulatory circuit composing positive-feedback loops with the Ras1-regulated Wor1 in a central position 67.

## RAS SIGNALLING AND *C. ALBICANS* BIOFILM FORMATION

Biofilms are structured communities of microorganisms which are attached to either a living or non-living surface. The cells are often encased within a matrix of self-made extracellular polymeric substance (EPS); this EPS is composed of DNA 69,70, lipids 69, proteins 69,71 and polysaccharides 69. Medically, biofilms are of particular importance because it is thought that a significant percentage of human microbial infections include biofilm formation 72,73,74. Moreover, cells which reside within biofilms have distinctive phenotypes compared to planktonic cells, for example, they exhibit increased resistance to antibiotic and antifungal drugs. The reasons for this increased resistance are complex but include the presence of an extracellular matrix reducing the ability of antimicrobial agents to reach the cells, metabolic differences (such as modulation of glycolysis, ergosterol biosynthesis and mitochondrial respiration) 75 inherent to biofilms and upregulation of efflux pumps 76. *C. albicans* biofilms are usually composed of a mixture of morphological forms; typically yeast, pseudohyphal and true hyphal cells are all present within a mature biofilm 6,77,78. The formation of a biofilm is the result of a very precise and complex series of events that are divided into distinct stages; attachment, initiation, maturation and dispersal. Biofilm formation is therefore complex and highly regulated with more than 1000 genes found to be upregulated during biofilm development 79.

Hyphal cells are important for the formation of *C.albicans* biofilms, one reason for this is that the expression of several cell surface adhesins, such as Hwp1 and Eap1, is increased during hyphal growth. These adhesins are required for the initial attachment phase of biofilm formation, and as a result it means Ras signalling is strongly linked to their development 80. This is highlighted by the finding that the hyphal-defective mutant *efg1*∆/∆ is unable to form biofilms 81. Rather than the true basal layer which wild-type *C. albicans *cells form, *efg1*∆/∆ mutants produce very few surface-attached cells. Despite this the surface-attached mutant cells do display resistance to both fluconazole and amphotericin B 81. These are important observations that suggest surface-adhesion is sufficient to induce an antifungal resistance response in biofilms 78.

The transcription factor Bcr1, which is upregulated by Tec1 (Figure 1) is an important regulator of *C. albicans *biofilm formation. The *bcr1*∆/∆ mutant is unable to form biofilms and also cannot switch to hyphal growth under certain conditions. However, when present within mixed biofilms formed using wild-type cells, *bcr1*∆/∆ mutant cells can form hyphae 82. Interestingly, *bcr1*∆/∆ hyphal cells themselves are unable to adhere to surfaces and initiate biofilm formation. Bcr1 upregulates a number of genes which encode cell wall proteins, including the adhesins Als1, Als3, and Hwp1 79. It is likely therefore that hyphal associated cell wall composition is crucial for biofilm formation. Ras1-Cyr1-PKA signalling is important in this respect as it regulates the expression of adhesins, such as Als1, via its control of the activity of the key transcription factors Efg1, Tec1 and Bcr1 79. cAMP/PKA signalling is also likely to impact upon biofilm formation with respect to CO_2_ levels. For example, a local accumulation of CO_2_ within *C. albicans* colonies was sufficient to induce filamentous growth 31. Although the precise roles have yet to be determined, it will be interesting to examine how CO_2_ signalling contributes to biofilm establishment *in vivo*. The final stage of biofilm development is the dispersal stage in which a mature biofilm begins to ‘throw’ fragments off in order to establish additional biofilms elsewhere 78. The transcription factor Nrg1, whose degradation is inhibited by the *C. albicans* quorum sensing molecule farnesol, has been shown to promote biofilm cell dispersion 83. As Ras activation can influence Nrg1 levels, and as farnesol has been shown to promote Ras1 degradation it will be of interest to investigate how Ras signalling influences the biofilm dispersion process.

## RAS SIGNALLING AND VIRULENCE IN *CRYPTOCOCCUS NEOFORMANS*

The prototypical species in the *Cryptococcus* genus is *C. neoformans *which is an encapsulated, pleomorphic yeast 84. Similar to *C. albicans, C. neoformans *is an opportunistic human pathogen with infection primarily being associated with a compromised immune system; cryptococcal infections (cryptococcosis) are a particular a problem amongst HIV/AIDS patients 85. The Centers for Disease Control and Prevention estimates the number of deaths attributed to cryptococcal meningitis in HIV/AIDS patients is as high as 181000 per year 86. *C. neoformans* possesses two *RAS* genes, denoted *RAS1* and *RAS2*, which encode the Ras1 and Ras2 proteins respectively 87. The Ras1 protein is highly conserved: it is a homolog of the traditional Ras proteins in mammalian cells such as *H-RAS* 84 and has been demonstrated to be essential for multiple processes in *C. neoformans* including growth at 37^°^C (Ras1 is not needed for growth at 30^°^C), mating, agar adherence and filamentation 88,889. Due to its crucial role in these processes, particularly thermotolerance, Ras1 is considered as an important virulence factor. Indeed, the ∆*ras*1 mutant strain is avirulent in a rabbit model of cryptococcal meningitis 88. Moreover, introduction of a dominant active *RAS1* allele (*RAS1*^Q67L^), which was constructed by introducing a point mutation in the active site of the GTPase domain of Ras1, resulted in significant increases in filamentation and agar invasion of haploid *C. neoformans* cells 88. These are two properties which are very important for virulence, supporting the conclusion that Ras1 signalling is vital to the pathogenicity of *C. neoformans*. It is important to note that in other model systems, including *Caenorhabditis elegans* 90 and *Drosophila melanogaster* 91, the ∆*ras1* mutant strain exhibited decreased virulence at lower temperatures. This implies that, at least for non-mammalian model systems, Ras1 signalling may have significant functions in the pathogenicity of *C. neoformans *besides thermotolerance.

**Figure 2:**
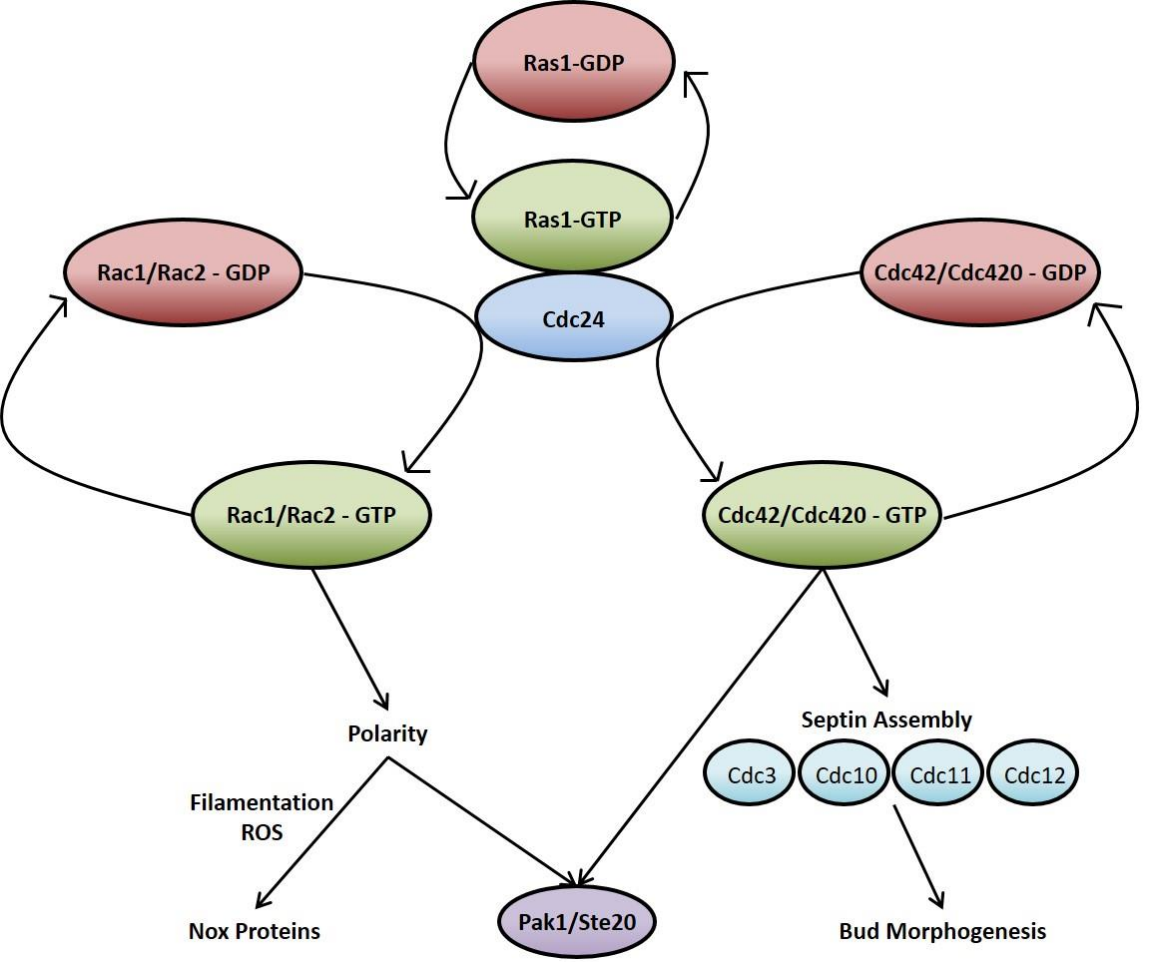
FIGURE 2: Ras1 signals via Cdc42 and Rac proteins to regulate many processes within the *C. neoformans* cell. Upon activation Ras1 associates with Cdc24 and subsequently activates the Rho-type GTPases Rac1/Rac2 and Cdc42/Cdc420. These proteins are involved in multiple cellular processes such as cell cycle progression, mating and morphogenesis.

Ras signalling in *C. neoformans*, much like in mammalian cells, acts through and/or in concert with other Rho-type GTPases such as Cdc42 and Rac 89,92,93,94,95. As is the case with *RAS*, *C. neoformans *possesses duplicate copies of *CDC*42 (*CDC*42 and *CDC*420) 92 and *RAC* (*RAC*1 and *RAC*2) 93. The overexpression of any of these genes overcomes the thermotolerance deficiencies of the ∆*ras*1 mutant, supporting a model whereby these GTPases act downstream of Ras1 (Figure 2) 89,5. At 37^°^C, ∆*ras*1 *C. neoformans *mutants amass a number of defects in cell cycle progression, specifically concerning cell polarisation and cytokinesis. While neither is required for viability when the organism is not under stress, the two Cdc42 paralogues are essential for septin organisation and effective cytokinesis at 37^°^C 92. Ras1 is also important in the activation of Cdc42 and Rac 94. Indeed, Cdc24 which is the reported GEF for Cdc42, undergoes a GTP-dependent physical interaction with Ras1 89,96. Once activated, Cdc42 organises the septin proteins Cdc3, Cdc10, Cdc11 and Cdc12, causing them to localise to the bud neck ready for cytokinesis 94. In addition to septin protein organisation, Ras1 signalling through Cdc42 is also necessary for normal bud morphology 94. Cdc42 appears to be the more important to *C. neoformans *virulence since it, and not Cdc420, is upregulated during temperature stress and is necessary for virulence in a mouse model of *Cryptococcus* infection 92. As previously mentioned, Ras1 is not required for growth of *C. neoformans *at 30^o^C. The reason for this appears to be due to basal levels of Cdc42 activity even in the absence of Ras1 signalling which is sufficient to allow proliferation at this lower temperature 92,94.

Although functional redundancy may exist between Cdc42 and Rac, the two Rac paralogues in *C. neoformans* are predominantly involved in cell polarisation and polarised growth; particularly in hyphal development when mating and in the transport of vesicles during the yeast phase of growth 93,95,97. The ∆*rac*1 mutant strain, while still able to grow at 37^o^C, displays a significant defect in haploid filamentation along with reduced mating 95. ∆*rac*1 and ∆*rac*2 mutant strains have increased yeast cell size 93, in keeping with the phenotype exhibited by ∆*ras*1 cells when grown at 37^°^C 88 as well as that shown by polarity mutants of the prototypical budding yeast *S. cerevisiae* 98. This highlights the important role Ras1 signalling through Rac1 and Rac2 plays in cell polarity and this precisely impacts hyphal development and yeast cell size.

As with *C. albicans*, the ability to sense CO_2_ is of critical importance to *C. neoformans*. Capsule biosynthesis, which is a major virulence characteristic, is increased by high CO_2_ levels and this is mediated by the *C. neoformans *adenylate cyclase Cac1 20. Importantly, it has been demonstrated that a fragment of CnCac1 can restore hyphal development in response to elevated CO_2_ within a *cyr1*∆/∆ *C. albicans* mutant. This implies the link between CO_2 _sensing and cAMP could be a general feature of pathogenic yeasts since *C. neoformans* and *C. albicans* are evolutionarily distantly related but their adenylate cyclases are functionally highly conserved 20.

The *C. neoformans* Ras2 protein plays separable roles to those of Ras1. For instance, ∆*ras*2 cells do not exhibit reduced virulence in a mouse model of cryptococcosis 87. Furthermore, ∆*ras*2 cells do not exhibit the same mating defects associated with the loss of *RAS1* 87. *RAS*2 is also found to be expressed at low levels compared to *RAS*1 and ∆*ras*2 mutants do not display any differences in growth or differentiation when compared to wild-type 87. However, it is worth noting that overexpression of *RAS*2 in a ∆*ras*1 mutant is able to rescue the mating defect and partially restores its growth at high temperature 87. Moreover, the double ∆*ras*1∆*ras*2 mutant has growth defects at all temperatures which are worse than with either single mutation alone 87. These findings suggest that Ras1 and Ras2 protein may have some overlapping functions in growth, mating and virulence that have yet to be properly defined.

## TARGETING RAS TO COMBAT YEAST PATHOGENESIS

Given its role in the virulence and pathogenic properties of multiple yeast (and other fungal) species, it would seem to have potential as anti-fungal drug target. Additionally, it has been shown that the manipulation of Ras signalling is an important control point in the activation of apoptosis in the budding yeast *S. cerevisiae* 99,100,101 and in *C. albicans* 102. These findings present the possibility that the pharmacological manipulation of Ras signalling may be useful in the induction of yeast cell death. Some current antifungals do exert an effect on fungal Ras signalling but this is indirect. For example, amphotericin B inserts into ergosterol-containing fungal membranes to form aqueous pores 103 which cause a local thinning of the bilayer. This thinning of the bilayer has been proposed to force lipid-anchored Ras proteins into sterol-rich lipid rafts, promoting its interactions with downstream proteins and thus activating signalling 104. Enhanced Ras signalling through pharmacological manipulation induces yeast apoptosis and oxidative damage via the cAMP-PKA pathway, indicated by the fact *S. cerevisiae* ∆*ras1, *∆*ras2*, ∆*tpk1*, ∆*tpk2* and ∆*tpk3* mutants have reduced amphotericin B-induced reactive oxygen species production and hence are unaffected by the lethal effect of amphotericin B 105. Other fungicides such as miconazole and ciclopirox have been reported to cause fungal cell death via a similar mechanism 105.

Ras proteins have proven difficult to directly target pharmacologically and because the GTPase domain is very highly conserved any attempt to target a fungal Ras may result in unfavourable side effects upon the host. Encouragingly a recent discovery does suggest that fungal Ras proteins may be targetable after all. A recent study demonstrated that the RasA protein of the human fungal pathogen *Aspergillus fumigatus* possesses a short N-terminal tail domain which is missing from Ras homologs in higher eukaryotes 106. This domain takes the form of a short length of amino acid residues which terminates in an arginine and has been dubbed the invariant arginine domain (IRD). Through sequence alignment, it has been reported that the IRD is present in many different fungal pathogens including *C. albicans *and *C. neoformans*, as well as non-pathogenic fungi such as *S. cerevisiae *and *S. pombe* 106. Mutation of the *A. fumigatus* RasA IRD decreased the activation of PKA as well as reducing the interaction of RasA with Cdc42 106 which controls polarity and thus is essential for normal growth and cell division 107. In line with this, mutational analyses confirmed that the IRD is necessary for polarised morphogenesis, a characteristic strongly linked with pathogenesis in *A. fumigatus*, and asexual development 106. These findings point towards the possibility of designing drugs to specifically target the IRD as a new Ras-based pan-antifungal therapy. As an extension of this it may also be possible to target regions within the C-terminal variable domain of fungal Ras proteins to modulate its activity.

## CONCLUSIONS 

Ras signalling has proven to be an important component of the growth and adaptability of fungal cells, as is indeed the case in higher eukaryotes. It is also crucial to the virulence and pathogenic properties of fungal species. Ras signalling therefore represents an interesting therapeutic target if fungal specific targets can be found. This may be particularly effective when used in combination with other antifungal agents. A full understanding of the Ras signalling network and its effectors will be required to achieve this aim. As an example, it may be possible to identify a small molecule that can activate fungal Ras in *C. albicans*. This may, at first glance, seem an unlikely intervention given the role of Ras signalling in promoting growth. However, as we now also know that the activation of Ras sensitizes cells to cell death, such an approach may prove particularly effective when used in combination with existing antifungals. Given the rise of antifungal resistance and our limited number of existing targets such "knowledge based" approaches will doubtless prove crucial in future therapy development.

## References

[1] Bourne HR, Sanders DA, McCormick F (1991). The GTPase superfamily: conserved structure and molecular mechanism.. Nature.

[2] Boguski MS, McCormick F (1993). Proteins regulating Ras and its relatives.. Nature.

[3] Campbell PM, Der CJ (2004). Oncogenic Ras and its role in tumor cell invasion and metastasis.. Semin Cancer Biol.

[4] Berman J, Sudbery PE (2002). Candida albicans: a molecular revolution built on lessons from budding yeast.. Nat Rev Genet.

[5] Ganguly S, Mitchell AP (2011). Mucosal biofilms of Candida albicans.. Curr Opin Microbiol.

[6] Douglas LJ (2003). Candida biofilms and their role in infection.. Trends Microbiol.

[7] Finkel JS, Mitchell AP (2011). Genetic control of Candida albicans biofilm development.. Nat Rev Microbiol.

[8] Talpaert MJ, Balfour A, Stevens S, Baker M, Muhlschlegel FA, Gourlay CW (2015). Candida biofilm formation on voice prostheses.. J Med Microbiol.

[9] Kim J, Sudbery P (2011). Candida albicans, a major human fungal pathogen.. J Microbiol.

[10] Kibbler CC, Seaton S, Barnes RA, Gransden WR, Holliman RE, Johnson EM, Perry JD, Sullivan DJ, Wilson JA (2003). Management and outcome of bloodstream infections due to Candida species in England and Wales.. J Hosp Infect.

[11] Koh AY, Köhler JR, Coggshall KT, Van Rooijen N, Pier GB (2008). Mucosal Damage and Neutropenia Are Required for Candida albicans Dissemination.. PLoS Pathog.

[12] Mikulska M, Del Bono V, Ratto S, Viscoli C (2012). Occurrence, presentation and treatment of candidemia.. Expert Rev Clin Immunol.

[13] Blot SI, Vandewoude KH, Hoste EA, Colardyn FA (2002). Effects of nosocomial candidemia on outcomes of critically ill patients.. Am J Med.

[14] Laprade L, Boyartchuk VL, Dietrich WF, Winston F (2002). Spt3 plays opposite roles in filamentous growth in Saccharomyces cerevisiae and Candida albicans and is required for C.. albicans virulence. Genetics.

[15] Braun BR, Head WS, Wang MX, Johnson AD (2000). Identification and characterization of TUP1-regulated genes in Candida albicans.. Genetics.

[16] Uhl MA (2003). Haploinsufficiency-based large-scale forward genetic analysis of filamentous growth in the diploid human fungal pathogen C.. lbicans. EMBO J.

[17] Leberer E, Harcus D, Dignard D, Johnson L, Ushinsky S, Thomas DY, Schröppel K (2001). Ras links cellular morphogenesis to virulence by regulation of the MAP kinase and cAMP signalling pathways in the pathogenic fungus Candida albicans.. Mol Microbiol.

[18] Bell WM, Chaffin WL (1983). Effect of yeast growth conditions on yeast-mycelial transition in Candida albicans.. Mycopathologia.

[19] Shapiro RS, Uppuluri P, Zaas AK, Collins C, Senn H, Perfect JR, Heitman J, Cowen LE (2009). Hsp90 Orchestrates Temperature-Dependent Candida albicans Morphogenesis via Ras1-PKA Signaling.. Curr Biol.

[20] Klengel T, Liang W-J, Chaloupka J, Ruoff C, Schröppel K, Naglik JR, Eckert SE, Mogensen EG, Haynes K, Tuite MF, Levin LR, Buck J, Mühlschlegel FA (2005). Fungal adenylyl cyclase integrates CO2 sensing with cAMP signaling and virulence.. Curr Biol.

[21] Mattia E, Carruba G, Angiolella L, Cassone A (1982). Induction of Germ Tube Formation by N-Acetyl-D- Glucosamine in Candida albicans: Uptake of Inducer and Germinative Response.. J Bacteriol.

[22] Feng Q, Summers E, Guo B, Fink G (1999). Ras signaling is required for serum-induced hyphal differentiation in Candida albicans.. J Bacteriol.

[23] Hoyer LL, Payne TL, Bell M, Myers AM, Scherer S (1998). Candida albicans ALS3 and insights into the nature of the ALS gene family.. Curr Genet.

[24] Staab JF, Bradway SD, Fidel PL, Sundstrom P (1999). Adhesive and mammalian transglutaminase substrate properties of Candida albicans Hwp1.. Science.

[25] Bailey DA, Feldmann PJ, Bovey M, Gow NA, Brown AJ (1996). The Candida albicans HYR1 gene, which is activated in response to hyphal development, belongs to a gene family encoding yeast cell wall proteins.. J Bacteriol.

[26] Zheng X, Wang Y, Wang Y (2004). Hgc1, a novel hypha-specific G1 cyclin-related protein regulates Candida albicans hyphal morphogenesis.. EMBO J.

[27] Gow NAR, van de Veerdonk FL, Brown AJP, Netea MG (2011). Candida albicans morphogenesis and host defence: discriminating invasion from colonization.. Nat Rev Microbiol.

[28] Bockmühl DP, Ernst JF (2001). A potential phosphorylation site for an A-Type kinase in the Efgl regulator protein contributes to hyphal morphogenesis of Candida albicans.. Genetics.

[29] Robertson LS, Causton HC, Young RA, Fink GR (2000). The yeast A kinases differentially regulate iron uptake and respiratory function.. Proc Natl Acad Sci U S A.

[30] Pohlers S, Martin R, Kruger T, Hellwig D, Hanel F, Kniemeyer O, Saluz HP, van Dijck P, Ernst JF, Brakhage A, Muhlschlegel FA, Kurzai O (2017). Lipid Signaling via Pkh1/2 Regulates Fungal CO2 Sensing through the Kinase Sch9.. Am Soc Microbiol.

[31] Hall RA, de Sordi L, MacCallum DM, Topal H, Eaton R, Bloor JW, Robinson GK, Levin LR, Buck J, Wang Y, Gow NAR, Steegborn C, Mühlschlegel FA (2010). CO2 acts as a signalling molecule in populations of the fungal pathogen Candida albicans.. PLoS Pathog.

[32] Xu X-L, Lee RTH, Fang H-M, Wang Y-M, Li R, Zou H, Zhu Y, Wang Y (2008). Bacterial Peptidoglycan Triggers Candida albicans Hyphal Growth by Directly Activating the Adenylyl Cyclase Cyr1p.. Cell Host Microbe.

[33] Maidan M, De Rop L, Serneels J, Exler S, Rupp S, Tournu H, Thevelein J, Van Dijck P (2005). The G protein-coupled receptor Gpr1 and the Ga protein Gpa2 act through the cAMP-protein kinase A pathway to induce morphogenesis in Candida albicans.. Mol Biol Cell.

[34] Ivey FD, Hoffman CS (2005). Direct activation of fission yeast adenylate cyclase by the Gpa2 Galpha of the glucose signaling pathway.. PNAS.

[35] Hollomon JM, Grahl N, Willger SD, Koeppen K, Hogan DA (2016). Global Role of Cyclic AMP Signaling in pH-Dependent Responses in Candida albicans.. mSphere.

[36] Hudson DA, Sciascia QL, Sanders RJ, Norris GE, Edwards PJB, Sullivan PA, Farley PC (2004). Identification of the dialysable serum inducer of germ-tube formation in Candida albicans.. Microbiology.

[37] Rolland F, De Winde JH, Lemaire K, Boles E, Thevelein JM, Winderickx J (2000). Glucose-induced cAMP signalling in yeast requires both a G-protein coupled receptor system for extracellular glucose detection and a separable hexose kinase-dependent sensing process.. Mol Microbiol.

[38] Goswami R, Dadhwal V, Tejaswi S, Datta K, Paul A, Haricharan RN, Banerjee U, Kochupillai NP (2000). Species-specific prevalence of vaginal candidiasis among patients with diabetes mellitus and its relation to their glycaemic status.. J Infect.

[39] Guggenheimer J, Moore PA, Rossie K, Myers D, Mongelluzzo MB, Block HM, Weyant R, Orchard T (2000). Insulin-dependent diabetes mellitus and oral soft tissue pathologies.. II. Prevalence and characteristics of Candida and candidal lesions. Oral Surgery, Oral Med Oral Pathol Oral Radiol Endodontology.

[40] Rodaki A, Bohovych IM, Enjalbert B, Young T, Odds FC, Gow NAR, Brown AJP (2009). Glucose Promotes Stress Resistance in the Fungal Pathogen Candida albicans.. Mol Biol Cell.

[41] Biswas K, Morschhäuser J (2005). The Mep2p ammonium permease controls nitrogen starvation-induced filamentous growth in Candida albicans.. Mol Microbiol.

[42] Liu H, Köhler J, Fink GR (1994). Suppression of hyphal formation in Candida albicans by mutation of a STE12 homolog.. Science.

[43] Köhler JR, Fink GR (1996). Candida albicans strains heterozygous and homozygous for mutations in mitogen-activated protein kinase signaling components have defects in hyphal development.. Proc Natl Acad Sci U S A.

[44] Leberer E, Harcus D, Broadbent I, Clark KL, Dignard D, Ziegelbauer K, Schmidt A, Gow NAR, Brown AJP, Thomas DY (1996). Signal transduction through homologs of the Ste20p and Ste7p protein kinases can trigger hyphal formation in the pathogenic fungus Candida albicans.. Proc Natl Acad Sci U S A.

[45] Csank C, Schroppel K, Leberer E, Harcus D, Mohamed O, Meloche S, Thomas DY, Whiteway M (1998). Roles of the Candida albicans Mitogen-Activated Protein Kinase Homolog, Cek1p, in Hyphal Development and Systemic Candidiasis†.. Infect Immun.

[46] Sanchez-Martinez C, Perez-Martin J (2002). Gpa2, a G-Protein alpha Subunit Required for Hyphal Development in Candida albicans.. Eukaryot Cell.

[47] Zhu Y, Fang HM, Wang YM, Zeng GS, Zheng X De, Wang Y (2009). Ras1 and Ras2 play antagonistic roles in regulating cellular cAMP level, stationary-phase entry and stress response in Candida albicans.. Mol Microbiol.

[48] Braun BR, Johnson AD (1997). Control of Filament Formation in Candida albicans by the Transcriptional Repressor TUP1.. Science.

[49] Murad AMA, Leng P, Straffon M, Wishart J, Macaskill S, Maccallum D, Schnell N, Talibi D, Marechal D, Tekaia F, Enfert C, Gaillardin C, Odds FC, Brown AJP (2001). NRG1 represses yeast-hypha morphogenesis and hypha-specic gene expression in Candida albicans.. EMBO J.

[50] Braun BR, Kadosh D, Johnson AD (2001). NRG1, a repressor of filamentous growth in C.. lbicans, is down-regulated during filament induction. EMBO J.

[51] Khalaf RA, Zitomer RS (2001). The DNA binding protein Rfg1 is a repressor of filamentation in Candida albicans.. Genetics.

[52] Kadosh D, Johnson AD (2005). Induction of the Candida albicans Filamentous Growth Program by Relief of Transcriptional Repression: A Genome-wide Analysis.. Mol Biol Cell.

[53] Lu Y, Su C, Wang A, Liu H (2011). Hyphal development in Candida albicans requires two temporally linked changes in promoter chromatin for initiation and maintenance.. PLoS Biol.

[54] Hull CM, Raisner RM, Johnson AD (2000). Evidence for Mating of the "Asexual" Yeast Candida albicans in a Mammalian Host.. Science.

[55] Forche A, Alby K, Schaefer D, Johnson AD, Berman J, Bennett RJ (2008). The parasexual cycle in Candida albicans provides an alternative pathway to meiosis for the formation of recombinant strains.. PLoS Biol.

[56] Bennett RJ, Johnson AD (2003). Completion of a parasexual cycle in Candida albicans by induced chromosome loss in tetraploid strains.. EMBO J.

[57] Hickman MA, Zeng G, Forche A, Hirakawa MP, Abbey D, Harrison BD, Wang YM, Su CH, Bennett RJ, Wang Y, Berman J (2013). The "obligate diploid" Candida albicans forms mating-competent haploids.. Nature.

[58] Herskowitz I (1988). Life cycle of the budding yeast Saccharomyces cerevisiae.. Microbiol Rev.

[59] Miller MG, Johnson AD (2002). White-opaque switching in Candida albicans is controlled by mating-type locus homeodomain proteins and allows efficient mating.. Cell.

[60] Soll DR (1997). Gene regulation during high-frequency switching in Candida albicans.. Microbiology.

[61] Pujol C, Daniels KJ, Lockhart SR, Srikantha T, Radke JB, Geiger J, Soll DR (2004). The closely related species Candida albicans and Candida dubliniensis can mate.. Eukaryot Cell.

[62] Lockhart SR, Pujol C, Daniels KJ, Miller MG, Johnson AD, Pfaller MA, Soil DR (2002). In Candida albicans, white-opaque switchers are homozygous for mating type.. Genetics.

[63] Tsong AE, Miller MG, Raisner RM, Johnson AD (2003). Evolution of a Combinatorial Transcriptional Circuit: A Case Study in Yeasts.. Cell.

[64] Huang G, Wang H, Chou S, Nie X, Chen J, Liu H (2006). Bistable expression of WOR1, a master regulator of white-opaque switching in Candida albicans.. Proc Natl Acad Sci.

[65] Huang G, Srikantha T, Sahni N, Yi S, Soll DR (2009). CO2 Regulates White-Opaque Switching in Candida albicans.. Curr Biol.

[66] Huang G, Yi S, Sahni N, Daniels KJ, Srikantha T, Soll DR (2010). N-acetylglucosamine induces white to opaque switching, a mating prerequisite in Candida albicans.. PLoS Pathog.

[67] Zordan RE, Miller MG, Galgoczy DJ, Tuch BB, Johnson AD (2007). Interlocking transcriptional feedback loops control white-opaque switching in Candida albicans.. PLoS Biol.

[68] Lohse MB, Hernday AD, Fordyce PM, Noiman L, Sorrells TR, Hanson-Smith V, Nobile CJ, DeRisi JL, Johnson AD (2013). Identification and characterization of a previously undescribed family of sequence-specific DNA-binding domains.. Proc Natl Acad Sci.

[69] Zarnowski R, Westler WM, Lacmbouh A, Marita JM, Bothe JR, Bernhardt J, Sahraoui AL, Fontaine J, Sanchez H, Hatfield RD, Ntambi JM, Nett JE, Mitchell AP, Andes R (2014). Novel Entries in a Fungal Biofilm Matrix Encyclopedia.. MBio.

[70] Martins M, Uppuluri P, Thomas DP, Cleary IA, Henriques M, Lopez-Ribot JL, Oliveira R (2014). Presence of extracellular DNA in the Candida albicans biofilm matrix and its contribution to biofilms.. Mycopathologia.

[71] Thomas DP, Bachmann SP, Lopez-Ribot JL (2006). Proteomics for the analysis of the Candida albicans biofilm lifestyle.. Proteomics.

[72] Costerton JW, Stewart PS, Greenberg EP (1999). Bacterial biofilms: a common cause of persistent infections.. Science (80- ).

[73] Donlan RM (2001). Biofilm formation: a clinically relevant microbiological process.. Clin Infect Dis.

[74] Donlan RM (2001). Biofilms and Device-Associated Infections.. Emerg Infect Dis.

[75] Verma-Gaur J, Qu Y, Harrison PF, Lo TL, Quenault T, Dagley MJ, Bellousoff M, Powell DR, Beilharz TH, Traven A (2015). Integration of Posttranscriptional Gene Networks into Metabolic Adaptation and Biofilm Maturation in Candida albicans.. PLoS Genet.

[76] Fanning S, Mitchell AP (2012). Fungal biofilms.. PLoS Pathog.

[77] Ramage G, Saville SP, Thomas DP (2005). Candida Biofilms: an Update.. Am Soc Microbiol.

[78] Chandra J, Kuhn DM, Mukherjee PK, Hoyer LL, McCormick T, Mahmoud A, Cormick TMC, Ghannoum MA (2001). Biofilm formation by the fungal pathogen Candida albicans: development, architecture, and drug resistance.. J Bacteriol.

[79] Nobile CJ, Fox EP, Nett JE, Sorrells TR, Mitrovich QM, Hernday AD, Tuch BB, Andes DR, Johnson AD (2012). A Recently Evolved Transcriptional Network Controls Biofilm Development in Candida albicans.. Cell.

[80] Desai J, Mitchell A (2015). Candida albicans biofilm development and its genetic control.. Microbiol Spectr.

[81] Ramage G, VandeWalle K, López-Ribot JL, Wickes BL (2002). The filamentation pathway controlled by the Efg1 regulator protein is required for normal biofilm formation and development in Candida albicans.. FEMS Microbiol Lett.

[82] Nobile CJ, Mitchell AP (2005). Regulation of cell-surface genes and biofilm formation by the C.. albicans transcription factor Bcr1p. Curr Biol.

[83] Lu Y, Su C, Unoje O, Liu H (2014). Quorum sensing controls hyphal initiation in Candida albicans through Ubr1-mediated protein degradation.. Proc Natl Acad Sci.

[84] Fortwendel J (2012). Ras-mediated signal transduction and virulence in human pathogenic fungi.. Fungal Genomics Biol.

[85] Park B, Wannemuehler K, Marston B, Govender N, Pappas P, Chiller T (2009). Estimation of the current global burden of cryptococcal meningitis among persons living with HIV/AIDS.. AIDS.

[86] Center for Disease Control and Prevention (2017) Global Fungal Diseases.. https://www.cdc.gov/fungal/global/index.htm.

[87] Waugh MS, Nichols CB, DeCesare CM, Cox GM, Heitman J, Alspaugh JA (2002). Ras1 and Ras2 contribute shared and unique roles in physiology and virulence of Cryptococcus neoformans.. Microbiology.

[88] Alspaugh JA, Cavallo LM, Perfect JR, Heitman J (2000). RAS1 regulates filamentation, mating and growth at high temperature of Cryptococcus neoformans.. Mol Microbiol.

[89] Nichols CB, Perfect ZH, Alspaugh JA (2007). A Ras1-Cdc24 signal transduction pathway mediates thermotolerance in the fungal pathogen Cryptococcus neoformans.. Mol Microbiol.

[90] Mylonakis E, Ausubel FM, Perfect JR, Heitman J, Calderwood SB (2002). Killing of Caenorhabditis elegans by Cryptococcus neoformans as a model of yeast pathogenesis.. Proc Natl Acad Sci U S A.

[91] Apidianakis Y, Rahme LG, Heitman J, Ausubel FM, Calderwood SB, Mylonakis E (2004). Challenge of Drosophila melanogaster with Cryptococcus neoformans and role of the innate immune response.. Eukaryot Cell.

[92] Ballou ER, Nichols CB, Miglia KJ, Kozubowski L, Alspaugh JA (2010). Two CDC42 paralogues modulate Cryptococcus neoformans thermotolerance and morphogenesis under host physiological conditions.. Mol Microbiol.

[93] Ballou ER, Selvig K, Narloch JL, Nichols CB, Alspaugh JA (2013). Two Rac paralogs regulate polarized growth in the human fungal pathogen Cryptococcus neoformans.. Fungal Genet Biol.

[94] Ballou ER, Kozubowski L, Nichols CB, Alspaugh JA (2013). Ras1 Acts through Duplicated Cdc42 and Rac Proteins to Regulate Morphogenesis and Pathogenesis in the Human Fungal Pathogen Cryptococcus neoformans.. PLoS Genet.

[95] Vallim MA, Nichols CB, Fernandes L, Cramer KL, Alspaugh JA (2005). A rac homolog functions downstream of Ras1 to control hyphal differentiation and high-temperature growth in the pathogenic fungus Cryptococcus neoformans.. Eukaryot Cell.

[96] Kozubowski L, Heitman J (2010). Septins enforce morphogenetic events during sexual reproduction and contribute to virulence of Cryptococcus neoformans.. Mol Microbiol.

[97] Shen G, Zhou E, Andrew Alspaugh J, Wanga P (2012). Wsp1 is downstream of Cin1 and regulates vesicle transport and actin cytoskeleton as an effector of Cdc42 and Rac1 in Cryptococcus neoformans.. Eukaryot Cell.

[98] Johnson JM, Jin M, Lew DJ (2011). Symmetry breaking and the establishment of cell polarity in budding yeast.. Curr Opin Genet Dev.

[99] Gourlay CW, Ayscough KR (2005). Identification of an upstream regulatory pathway controlling actin-mediated apoptosis in yeast.. J Cell Sci.

[100] Gourlay CW, Ayscough KR (2006). Actin-induced hyperactivation of the Ras signaling pathway leads to apoptosis in Saccharomyces cerevisiae.. Mol Cell Biol.

[101] Leadsham JE, Miller K, Ayscough KR, Colombo S, Martegani E, Sudbery P, Gourlay CW (2009). Whi2p links nutritional sensing to actin-dependent Ras-cAMP-PKA regulation and apoptosis in yeast.. J Cell Sci.

[102] Phillips AJ, Crowe JD, Ramsdale M (2006). Ras pathway signaling accelerates programmed cell death in the pathogenic fungus Candida albicans.. Proc Natl Acad Sci U S A.

[103] Brajtburg J, Bolard J (1996). Carrier Effects on Biological Activity of Amphotericin B.. Clin Microbiol Rev.

[104] Cohen BE (2016). The role of signaling via aqueous pore formation in resistance responses to amphotericin B.. Antimicrob Agents Chemother.

[105] Belenky P, Camacho D, Collins JJ (2013). Fungicidal Drugs Induce a Common Oxidative-Damage Cellular Death Pathway.. Cell Rep.

[106] Al Abdallah Q, Norton TS, Hill AM, LeClaire LL, Fortwendel JR (2016). A Fungus-Specific Protein Domain Is Essential for RasA-Mediated Morphogenetic Signaling in Aspergillus fumigatus.. mSphere.

[107] Adams A, Johnson D, Longnecker R, Sloat B, Pringle J (1990). CDC42 and CDC43, two additional genes involved in budding and the establishment of cell polarity in the yeast Saccharomyces cerevisiae.. J Cell Biol.

